# Risk Factors for Iliopsoas Impingement Following Total Hip Arthroplasty: A Systematic Review

**DOI:** 10.3390/jcm14186376

**Published:** 2025-09-10

**Authors:** Marco Minelli, Vincenzo Longobardi, Alessandro Del Monaco, Alessio D’Addona, Pierangelo Za, Federico Della Rocca, Mattia Loppini

**Affiliations:** 1 Department of Biomedical Sciences, Humanitas University, Via Rita Levi Montalcini 4, Pieve Emanuele, 20072 Milan, Italy; marcomariaminelli@gmail.com (M.M.); mattia.loppini@hunimed.it (M.L.); 2 IRCCS Humanitas Research Hospital, Via Manzoni 56, Rozzano, 20089 Milan, Italy; alessio.daddona@humanitas.it (A.D.); federico.della_rocca@humanitas.it (F.D.R.); 3 Operative Research Unit of Orthopaedic and Trauma Surgery, Fondazione Policlinico Universitario Campus Bio-Medico, Via Álvaro del Portillo, 00128 Roma, Italy; alessandro.delmonaco@unicampus.it; 4 Research Unit of Orthopaedic and Trauma Surgery, Department of Medicine and Surgery, Università Campus Bio-Medico di Roma, Via Álvaro del Portillo, 00128 Roma, Italy; 5 Department of Orthopaedic Surgery, GVM Care and Research–Città di Lecce Hospital, 73100 Lecce, Italy

**Keywords:** iliopsoas muscle, impingement syndrome, hip joint, arthroplasty, replacement, hip, groin, risk factors

## Abstract

**Background:** Iliopsoas impingement (IPI) is an increasingly recognized cause of persistent groin pain following total hip arthroplasty (THA), often resulting from mechanical conflict between the iliopsoas tendon and the anterior rim of the acetabular component. Despite its clinical relevance, risk factors contributing to IPI remain poorly defined. **Methods:** A systematic search of PubMed, Embase, Scopus, and the Cochrane Library was conducted according to PRISMA guidelines. Studies were eligible if they evaluated adult patients undergoing primary THA and reported at least one risk factor associated with IPI. Only studies with a clearly defined clinical diagnosis of IPI were included. Data extraction and risk of bias assessments were performed independently by two reviewers. Risk of bias in each study was assessed through the Newcastle-Ottawa Scale. **Results:** Twelve observational studies met the inclusion criteria. Diagnosis of IPI was based on clinical symptoms of anterior groin pain exacerbated by hip flexion; 9 studies confirmed diagnosis with anesthetic injections. Key surgical risk factors included anterior cup prominence (ORs 1.16–35.20), oversized cups (cup-to-head ratio > 1.2, OR = 5.39, or ≥6 mm difference, OR = 26.00), decreased cup inclination, collared stem protrusion (OR = 13.89), and acetabular screw protrusion > 6.4 mm. Patient-specific risk factors included female sex (ORs 2.56, 2.79), higher BMI (OR = 1.07), younger age, previous hip arthroscopy (OR = 9.60) and spinal fusion (OR = 4.60). The anterolateral approach was also associated with higher IPI risk when compared to the posterior approach (OR = 4.20). **Conclusions**: IPI after THA is a multifactorial complication influenced by modifiable surgical variables and patient-specific anatomy. Careful preoperative planning, precise implant positioning, and attention to individual risk factors are essential to reduce IPI incidence and improve outcomes.

## 1. Introduction

Total hip arthroplasty (THA) is a widely performed procedure with rising global prevalence [1,2]. As its use expands, so does the importance of recognizing complications such as iliopsoas impingement (IPI), a frequently underdiagnosed cause of postoperative groin pain [3]. IPI typically results from mechanical irritation of the iliopsoas tendon as it passes over the anterior rim of the acetabular component [3]. The reported prevalence of IPI after THA ranges from 0.4% to 8.3% [4,5].

The iliopsoas tendon is formed by the confluence of the psoas major and iliacus muscles, which descend from the lumbar spine and iliac fossa, respectively, and merge to insert on the lesser trochanter of the femur [6,7]. As it travels anterior to the hip joint, the tendon passes directly over the iliopectineal eminence and the anterior rim of the native acetabulum [6,7].

In this scenario, the etiology of IPI following total hip arthroplasty is multifactorial, involving both patient-specific and surgical variables. Several studies have implicated factors such as excessive anterior cup prominence, reduced anteversion, oversized acetabular components, and patient-specific anatomical variations [8,9]. Additionally, different surgical approaches and implant designs may influence the risk of developing iliopsoas impingement by altering the anatomical relationship between the iliopsoas tendon and the prosthetic components [3,10]. Although relatively uncommon, IPI can lead to significant functional impairment and dissatisfaction, sometimes necessitating further interventions including corticosteroid injections, tendon release, or revision arthroplasty [11]. However, no consensus has been reached regarding which risk factors are most predictive or preventable.

This systematic review aims to synthesize the current evidence on risk factors associated with IPI following THA. Specifically, we sought to address the following research questions (RQ):

- RQ1: What surgical factors (e.g., implant positioning, approach, component design) are associated with an increased risk of iliopsoas impingement after THA?
- RQ2: What patient-specific anatomical or demographic characteristics are associated with a higher incidence of IPI?
- RQ3: How consistently are these risk factors reported across clinical studies, and what is the strength of the available evidence?

By identifying consistent predictors across the literature, we aim to inform clinical decision-making, improve surgical planning, and reduce the incidence of this challenging complication.

## 2. Materials and Methods

### 2.1. Protocol and Registration

This systematic review was conducted in accordance with the Preferred Reporting Items for Systematic Reviews and Meta-Analyses (PRISMA) 2020 guidelines [12]. We searched the following electronic databases: PubMed/MEDLINE, Embase, Scopus, and the Cochrane Library. Additional sources included reference lists of included articles and relevant systematic reviews. All databases were last searched on the 15 April 2025. We did not search grey literature sources or preprint servers, which may have introduced publication bias by potentially excluding relevant unpublished or non-peer-reviewed studies. This systematic review was prospectively registered in the PROSPERO database (Registration ID: CRD420251121035).

### 2.2. Search Strategy and Selection Process

The search strategy combined controlled vocabulary (e.g., MeSH) and free-text terms. A sample search for PubMed included the following keywords: (iliopsoas OR psoas) AND (impingement OR tendinitis OR tendonitis OR groin pain) AND (total hip arthroplasty OR THA OR hip replacement). After removal of duplicates, two independent reviewers (M.M. and A.D.M) screened all titles and abstracts for eligibility using Rayyan (version 1.4.3, accessed on the 15 April 2025). Full texts of potentially relevant studies were then assessed independently. Prior to the screening process, a calibration exercise was conducted on a subset of studies. Inter-rater agreement between reviewers was assessed using Cohen’s kappa coefficient, demonstrating substantial agreement (κ = 0.84). Discrepancies during screening or data extraction were resolved through discussion or consultation with a third reviewer (V.L). No automation tools were used in the screening process.

### 2.3. Eligibility Criteria

Studies were eligible if they investigated adults (aged ≥ 18 years) who underwent primary total hip arthroplasty (THA) and subsequently developed symptoms of iliopsoas impingement (IPI). To be included, studies had to report at least one risk factor or predictor associated with IPI. We considered randomized controlled trials, prospective and retrospective cohort studies, case–control studies, and case series with ten or more patients. Only studies published in English were included. No date restrictions were applied during the literature search; all studies published up to the 15 April 2025 were considered for inclusion regardless of publication year. Excluded were studies on revision THA, hip resurfacing, animal or cadaveric studies, case reports, narrative reviews, editorials, conference abstracts, and studies lacking a clearly defined diagnosis of IPI or its associated risk factors.

### 2.4. Data Collection Process

Two reviewers (M.M. and V.L.) independently extracted data from all included studies using a standardized data extraction form. Discrepancies were resolved by consensus. When required, study authors were contacted to clarify missing or unclear data. No automation tools were used in this process.

### 2.5. Data Items

The primary outcome was the presence of risk factors associated with iliopsoas impingement after THA. We also collected data on patient demographics (age, sex), surgical approach, implant type and positioning, diagnostic criteria used for IPI, and outcomes such as prevalence or treatment response. For studies with unclear variables, assumptions were minimized and only explicitly reported data were extracted.

### 2.6. Risk of Bias Assessment

Risk of bias was assessed independently by two reviewers (M.M. and V.L.). For observational studies, the Newcastle-Ottawa Scale (NOS) was used to evaluate selection, comparability, and outcome domains. Randomized trials, if included, were assessed using the Cochrane Risk of Bias 2 (RoB 2) tool (Cochrane, London, UK). Agreement between reviewers was excellent, with a Cohen’s Kappa coefficient of 0.91, indicating near-perfect inter-rater reliability. Disagreements were resolved by consensus. No automation tools were used.

### 2.7. Effect Measures

Effect measures used to quantify associations between risk factors and IPI included odds ratios (ORs), relative risks (RRs), and hazard ratios (HRs), as reported in individual studies. Where available, 95% confidence intervals (CIs) were also extracted.

### 2.8. Synthesis Methods

A qualitative synthesis was conducted to summarize identified risk factors. When studies provided sufficient and comparable data, we planned to conduct a random-effects meta-analysis. Heterogeneity was assessed using the I^2^ statistic and Chi-square test. If meta-analyses were feasible, results would be presented using forest plots. Data were synthesized by study design and population characteristics to preserve clinical and methodological consistency.

## 3. Results

### 3.1. Studies Characteristics

**Figure 1:**
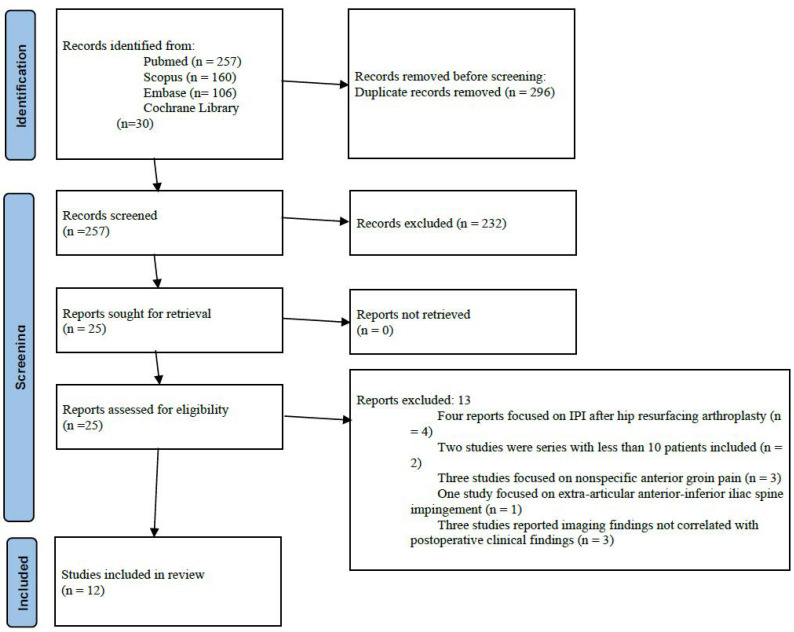
Flowchart of the selection process according to the PRISMA (Preferred Reporting Items for Systematic Reviews and Meta-Analyses) 2020 guidelines.

The initial search yielded a total of 257 potentially relevant articles after duplicates removal. After title and abstract selection, 232 articles were excluded. Four additional articles were excluded after full-text assessment: these studies included data on patients developing IPI after hip resurfacing. Two studies were excluded since these were case series with less than ten patients included. Three studies were excluded because they did not specifically assess iliopsoas tendinopathy, but rather included a broader population of patients with anterior groin pain of unspecified etiology. One study was excluded since it focused on extra-articular anterior-inferior iliac spine impingement. Three studies were excluded because they reported imaging findings suggestive of iliopsoas impingement but did not correlate these findings with postoperative clinical symptoms. Finally, 12 studies were analyzed [4,13,14,15,16,17,18,19,20,21,22,23] (Figure 1). Publication year ranged from 2014 to 2024. Nine studies were level of evidence III and three studies were level of evidence IV. Since all the included studies were observational studies, risk of bias in each study was assessed through the Newcastle-Ottawa Scale: eight studies achieved the maximum score of 9 stars, and the remaining four scored 8 stars, indicating low risk of bias and strong overall study quality (Table 1).

### 3.2. Patients Characteristics

Patient populations ranged from 23 to 1815 subjects per study. The number of hips in the included studies ranges from 23 to 2120. Mean age was reported in 10 studies [4,13,14,15,17,18,19,20,21,23] and ranged from 37.4 to 68.8 years. Mean follow-up was reported in 8 studies [4,13,14,18,19,21,22,23] and ranged from 24.2 to 55.9 months. Mean BMI was reported in 9 studies [4,14,15,17,18,19,20,21,23] and ranged from 23.2 to 28.1 kg/m^2^. Surgical approach was reported in 11 studies (for a total of 6108 hips) [4,13,14,15,16,17,18,19,20,21,23]: posterolateral approach was chosen for 1107 hips (18.1%), direct anterior for 2899 THAs (47.5%), anterolateral for 2102 hips (34.4%). Patients’ characteristics are summarized in Table 2.

### 3.3. Diagnosis of Iliopsoas Impingement

In all the included studies, the diagnosis of iliopsoas impingement was based on clinical criteria, namely the presence of anterior groin pain exacerbated by active hip flexion (Table 3). However, diagnostic confirmation methods varied across the included studies. In nine of the included studies [4,13,14,15,17,18,19,20,21], the diagnosis of iliopsoas impingement was further supported by the use of local anesthetic injections, with symptomatic relief considered confirmatory. One study employed imaging findings (ultrasound and MRI) to confirm iliopsoas tendinopathy or iliopectineal bursitis [19]. The prevalence of iliopsoas impingement was reported in 11 studies [4,13,14,15,17,18,19,20,21,22,23] and was reported to range from 1.5% to 11%.

### 3.4. Risk Factors

#### 3.4.1. Surgical Factors

Anterior cup prominence or cup overhang was identified to be significantly associated with iliopsoas impingement [13,16,18,19,20,21,23]:

- Buller et al. [13] found any measurable anterior cup overhang on the false profile to be significantly associated with IPI, with an adjusted odds ratio (aOR) of 7.07 (95% CI 2.52–19.78; *p* < 0.001);
- accordingly, Park et al. [4] reported an aOR of 15.43 for antero-inferior cup prominence > 8 mm on lateral radiographs (95% CI 3.75–63.47; *p* = 0.002);
- Kobayashi et al. [18] observed sagittal and axial anterior cup protrusion to be significantly associated with symptomatic IPI, with an aOR of 1.77 (95% CI 1.29–2.41; *p* < 0.001) and 1.16 (95% CI 1.03–1.29; *p* = 0.007), respectively;
- similarly, Ueno et al. [20] recorded that an axial protrusion length of 12 mm (aOR, 17.13; 95% CI, 2.78–105.46; *p* = 0.002) and a sagittal protrusion length of 4 mm (aOR, 8.23; 95% CI, 1.44–46.94; *p* = 0.018) were independent predictors of symptomatic IPI;
- anterior cup protrusion was observed to be significantly associated with iliopsoas impingement by Marth et al. [21] (OR 35.20, 95% CI 10.53–117.73; *p* < 0.001), Zhu et al. [19] (*p* < 0.001), Hardwick-Morris et al. [16] (>6.5 mm, *p* = 0.024) and Tamaki et al. [23] (*p* = 0.001).

Oversized cups were also associated with IPI:

- Odri et al. [22] showed that a difference between implanted and native femoral head diameter ≥6 mm was significantly associated with IPI (aOR 26.00; 95% CI 6.30–108.00, *p* < 0.01);
- Kobayashi et al. [18] observed oversized cups to be associated with symptomatic iliopsoas impingement (aOR 1.26; 95% CI 0.89–1.66, *p* = 0.192).

Accordingly, Buller et al. [13] reported that a cup-to-head ratio >1.2 significantly increased the risk of developing iliopsoas impingement (aOR 5.39; *p* < 0.05).

As regards cup positioning, Odri et al. found significantly lower inclination angles in symptomatic patients (*p* = 0.03). With regard to acetabular screw positioning, Ueki et al. found that screw protrusion >6.4 mm was associated with iliopsoas tendinopathy and poorer outcomes (*p* < 0.001) [15]. Similarly, Zhu et al. [19] observed protruded acetabular screws to be associated with IPI (*p* < 0.001).

Ueno et al. reported that the anterolateral approach was associated with a significantly higher risk of IPI compared to the posterior approach (aOR 4.20, 95% CI 1.68–10.49, *p* = 0.002) [20].

Excessive stem anteversion (aOR 1.75, 95% CI 1.25–2.43, *p* = 0.001) and collar protrusion (aOR 13.89, 95% CI 3.14–62.50, *p* = 0.001) were identified to be independent predictors for IPI after cementless collared THA by Qiu et al. [15]. Moreover, patients with Dorr type C bone appeared to be at higher risk of developing IPI after cementless collared THA [15].

Leg lengthening was observed to be significantly associated with iliopsoas impingement by Park et al. [4] (OR 1.06, CI 1.03–1.11, *p* = 0.018) and by Zhu et al. [19] (*p* < 0.001).

#### 3.4.2. Patient-Related Factors

Several studies identified patient-specific characteristics that independently predicted IPI. Female sex was associated with higher IPI risk in multivariable analyses by Park et al. [19] (aOR 2.56; 95% CI 1.36–4.82; *p* = 0.012) and Buller et al. [13] (aOR 2.79; 95% CI 1.18–6.56 *p* = 0.020). Higher body mass index (BMI) was also found to be a significant predictor by Park et al. [19], with an aOR of 1.14 per unit increase (95% CI 1.07–1.21; *p* < 0.001). Verhaegen et al. [14] reported younger age (*p* < 0.001), history of spinal fusion (aOR 4.6; 95% CI 1.60–13.40, *p* = 0.016) and previous hip arthroscopy (OR 9.60, 95% CI 2.60–34.30, *p* = 0.002) to be significantly associated with increased IPI risk. Preoperative and postoperative hip flexion angles were observed to be significantly greater in patients with symptomatic IPI (*p* = 0.013 and *p* = 0.006, respectively) by Tamaki et al. [23]. Risk factors are summarized in Table 3 and Table 4.

## 4. Discussion

This systematic review aimed to identify and synthesize reported risk factors for iliopsoas impingement (IPI) following total hip arthroplasty (THA). Across the 12 included studies, several consistent and clinically relevant predictors were identified. These include modifiable surgical factors such as anterior cup prominence, oversized acetabular components, decreased cup inclination, acetabular screw protrusion and excessive leg lengthening, as well as patient-specific factors including higher body mass index (BMI), female sex, younger age, history of spinal fusion, and spinopelvic anatomy. To our knowledge, this is the first systematic review to comprehensively evaluate both surgical and anatomical risk factors for IPI using exclusively clinical cases of post-THA groin pain with clearly defined diagnostic criteria. As such, direct comparisons with prior systematic reviews are not feasible. Nevertheless, our findings align with individual observational studies that have reported significant associations between surgical variables and patient-specific factors and the risk of developing IPI.

Implant positioning was among the most frequently implicated surgical factors. Anterior cup prominence or overhang emerged as the most consistently reported risk factor for IPI across the included studies [13,16,18,19,20,21,23]. This finding is biomechanically intuitive: a protruding cup rim increases the likelihood of mechanical conflict with the iliopsoas tendon during hip flexion, especially in deep flexion or stair climbing. The iliopsoas works as the principal dynamic hip flexor, functioning within a pulley system comprising the anterior border of the acetabulum and gliding over the iliopectineal eminence and adjacent anterior structures during movement [6,7,11]. Indeed, oversizing of the acetabular component, particularly with a cup-to-native head size ratio >1.2 or an absolute difference ≥6 mm, was also associated with increased impingement risk [13,22]. Then, anterior overreaming during surgery can lead to an anterior wall defect and increased cup protrusion, causing symptomatic IPI [18,24]. Additionally, reduced cup inclination and anteversion can increase the risk of protrusion of the acetabular component, thus were identified as contributors to IPI [16,25]. These results support the principle that restoring native femoral head geometry and hip center of rotation and avoiding overstuffing the anterior hip space are crucial for minimizing soft tissue conflict. Biomechanical cadaveric studies have confirmed that anterior cup protrusion, decreased stem anteversion, and increased offset directly elevate iliopsoas surface pressure, particularly during hip extension [26]. Malpositioned components can alter the spatial relationship between the prosthetic cup and the overlying iliopsoas tendon, increasing mechanical friction. Zhu et al. suggested that excessive anteversion could be associated with higher rates of IPI [19]. High combined functional anteversion may cause the iliopsoas to function as an anterior stabilizer to the prosthetic joint causing overuse and irritation, or may lead to posterior prosthetic impingement that irritates the iliopsoas through repeated anterior micro-instability [13,19,27]. Indeed, the iliopsoas serves to reinforce the anterior capsule ligaments as the hip is extended [28].

Protruding acetabular screws (>6.4 mm) were identified by both Ueki et al. [15] and Zhu et al. [19] as mechanical contributors to IPI, highlighting the importance of screw trajectory and intraoperative verification of screw length whenever supplemental screws are used to enhance the initial fixation of cementless acetabular cups. This could be particularly relevant in dysplastic hips, where the inherent bone deficiency and surgical necessity for initial cup fixation with screws can lead to a higher frequency of protruded screws [19]. However, Zhu et al. [19] did not observe a higher incidence of postoperative iliopsoas tendonitis in the dysplastic hips group. Thus, even subtle technical decisions during surgery may have significant clinical consequences in the development of IPI. In this scenario, Verhaegen et al. also reported a higher incidence of psoas-related pain in patients with ceramic-on-ceramic (CoC) bearing surfaces compared to ceramic-on-polyethylene (CoP) [14]. However, the sources clarify that this observed association appears to be an indirect one, linked to patient selection criteria rather than a direct causal mechanism of the ceramic bearing itself, since the study found a strong correlation between younger age and CoC bearing [14].

Surgical approach has been shown to influence the risk of iliopsoas impingement (IPI) after total hip arthroplasty. In particular, Ueno et al. [20] found the anterolateral surgical approach to be associated with a significantly higher risk of IPI compared to the posterior approach. This could be secondary to anterior capsule disruption during capsulotomy, which removes a protective layer that normally separates the iliopsoas complex from the acetabular component: when this layer is compromised, the iliopsoas tendon can come into direct contact with the acetabular component, potentially leading to mechanical irritation [13,21]. Dora et al. described how during arthroscopic iliopsoas tenotomy the iliopsoas tendon could be visualized directly through a defect in the anterior neocapsule, where the anterior rim of the acetabular cup was exposed [26]. Moreover, a less anteverted acetabular component might be favored in anterior approaches for stability benefits [29,30].

Several studies examined the role of femoral component design and positioning. Qiu et al. [17] identified excessive stem anteversion, collar protrusion, and Dorr type C femurs as risk factors for IPI when using cementless collared stems. Indeed, not only excessive stem anteversion could lead to anterior microinstability [13,19,27], but may also cause the collar to overhang beyond the edge of the calcar, leading to impingement on the distal segment of the iliopsoas tendon at the lesser trochanter [17]. This again underscores the relevance of combined anteversion, but also the physical profile of the implant in influencing IPI risk. In fact, Dorr type C femurs often require larger stem sizes, and an increase in stem size can be associated with increased collar length and collar protrusion, thus increasing the likelihood of impingement [17].

Patient-specific anatomical factors also played a significant role. Park et al. [19] and Verhaegen et al. [14] found that female sex, higher BMI, younger age, and spinal fusion were all associated with an increased risk of IPI. Particularly, spinal fusion may rigidify the spinopelvic segment and impair pelvic adaptability during positional changes, thereby increasing anterior impingement during hip flexion [31]. The influence of spinopelvic stiffness is supported by biomechanical studies demonstrating that restricted posterior pelvic tilt limits acetabular anteversion in sitting, forcing excessive femoral hyperflexion and increasing iliopsoas-tendon contact with anterior hardware [32,33]. Tamaki et al. [23] further observed that both pre- and postoperative hip flexion angles were significantly greater in patients with IPI, suggesting that dynamic motion demands in certain anatomical or postural configurations augment mechanical tendon stress. Instead, female sex is consistently associated with an increased propensity or risk for IPI, since women generally have smaller native acetabular diameters compared to men, resulting in differences in acetabular diameter, anteversion, and depth of the psoas valley [34,35]. In order to accommodate a larger prosthetic femoral head and yield a better head-neck ratio, the acetabulum should be reamed to a larger size and this can increase the risk of cup overfitting and lead to a greater cup-to-native femoral head ratio in women, leading to anterior component overhang [13,21]. Moreover, a higher incidence of hip dysplasia in females may contribute to a greater degree of anterior acetabular component overhang, especially if the anterior wall of the acetabulum is deficient [34,35]. Higher BMI may contribute to IPI by increasing anterior soft tissue bulk, which can reduce the space available for iliopsoas tendon excursion and elevate friction against the acetabular component [36,37,38]. Additionally, altered hip flexion mechanics and technical challenges in achieving optimal cup positioning in obese patients may further increase the risk of tendon irritation [36,37,38].

The findings of this review have direct clinical relevance for surgeons performing THA. To minimize the risk of iliopsoas impingement, careful preoperative planning and intraoperative techniques are essential. Intraoperatively, careful reaming and implant positioning are essential to avoid anterior cup overhang, particularly by ensuring the cup remains flush with or slightly recessed from the anterior native acetabular rim, thereby minimizing the risk of iliopsoas impingement. Then, oversized cups should be avoided: cup-to-native femoral head ratio should not exceed 1.2 [13], and the implanted cup diameter should not be ≥6 mm larger than the native head [22]. For acetabular screws, protrusion beyond 6.4 mm has been associated with increased risk of IPI and should be carefully checked using intraoperative fluoroscopy or depth gauges [15]. In patients with additional risk factors such as high BMI, female sex, spinal fusion, previous hip arthroscopy or abnormal spinopelvic parameters, further intraoperative adjustments may be warranted to reduce anterior impingement. These include optimizing sagittal cup orientation based on pelvic tilt [39]. From a broader perspective, this review reinforces the multifactorial etiology of IPI and the need for a patient-specific, anatomically respectful approach to THA. Particular vigilance is advised in patients undergoing anterolateral approaches, since these have been independently associated with increased IPI risk.

This review followed PRISMA guidelines and included a comprehensive search strategy across multiple databases. Only studies with clinically confirmed diagnoses of IPI were included, increasing the specificity and relevance of findings. All included studies defined IPI by anterior groin pain exacerbated by hip flexion, and most employed confirmatory diagnostic injections, enhancing diagnostic consistency.

However, several limitations must be acknowledged. First, all included studies were observational (level III or IV evidence), limiting the strength of causal inference. Second, while diagnostic criteria were largely consistent, definitions of risk factors (e.g., “cup prominence” or “oversized components”) varied between studies, limiting comparability. Third, due to heterogeneity in study designs and outcome measures, a meta-analysis was not performed. Then, some potential risk factors such as activity level, tendon morphology, or subtle anatomical variants were not addressed in the current literature. Lastly, the variability in diagnostic confirmation methods across studies could have contributed to differences in reported prevalence: studies utilizing anesthetic injections or imaging for confirmation likely provided more specific diagnoses, while those relying solely on clinical symptoms may be at greater risk of diagnostic overlap or misclassification.

Prospective studies using standardized definitions and imaging protocols are needed to validate the risk factors identified in this review. Further research should explore the interaction between spinopelvic dynamics and impingement, and assess whether surgical planning tools could reduce the incidence of IPI. Long-term studies comparing implant types and bearing surfaces may also clarify the role of hardware-related factors in IPI development.

## 5. Conclusions

This systematic review identified key surgical and patient-specific risk factors for iliopsoas impingement (IPI) following total hip arthroplasty (THA). Anterior cup overhang and oversized components were the most consistent surgical predictors. Patient factors such as female sex, higher BMI, younger age, and spinal fusion also increased IPI risk. The anterolateral approach was associated with a higher incidence of IPI compared to the posterior approach.

While these findings suggest important trends, they should be interpreted with caution due to heterogeneity in study designs, diagnostic criteria, and predominantly observational data. Nonetheless, the results support the need for careful preoperative planning and implant positioning tailored to individual patient anatomy and biomechanics. Future high-quality prospective studies are warranted to confirm these associations and inform evidence-based surgical recommendations.

## Figures and Tables

**Table 1:** Newcastle-Ottawa quality assessment scale for cohort studies (★ = 1 point; ★★ = 2 points according to the Newcastle–Ottawa Scale scoring system).

Study Name	Selection 1: Representativeness of Exposed Cohort/Cases	Selection 2: Selection of Non-Exposed Cohort/Controls	Selection 3: Ascertainment of Exposure	Selection 4: Outcome Not Present at Baseline (Cohort)/Definition of Cases (Case–Control)	Comparability: Control for Confounders (Max 2 Stars)	Outcome 1: Assessment of Outcome/Exposure	Outcome 2: Follow-Up Long Enough/Same Method for Cases and Controls	Outcome 3: Adequacy of Follow-Up/Non-Response Rate	Total Stars (Max 9)	Notes
Buller et al.	**★**	**★**	**★**	**★**	**★★**	**★**	**★**	**★**	**9**	Retrospective study: Some detection bias possible (patients with pain more likely to return for follow-up). Potential recall bias for telephone follow-up. Single-institution, retrospective nature limits generalizability. Asymptomatic controls drawn from the same cohort of patients (same institution and time frame). Controlled for age, gender, native femoral head size by matching and multivariable regression, mean follow-up 56 months (>2 years minimum) is adequate
Hardwick-Morris et al.	**★**	**★**	**★**	**★**	**★★**	**★**	**★**	**★**	**9**	Retrospective study; Small cohort (23 patients); clear inclusion and diagnostic criteria; controls (n = 23) randomly selected from the same THA population and external referrals, matched for inclusion/exclusion criteria; logistic regression adjusting for cup prominence, standing mean impingement and standing maximum impingement, Same 3D modeling and diagnostic pathway applied to both cases and controls
Kobayashi et al.	**★**	**★**	**★**	**★**	**★★**	**★**	**★**	**★**	**9**	Retrospective study; standardized validated methods for instrumental outcomes, use of contralateral (non-affected side) as control, Logistic and multivariable regression adjusted for several factors (COR, cup protrusion length, acetabular offset, etc.), Low attrition: 138/178 eligible patients included (lost/deaths documented); reasons for exclusions clearly stated, median follow-up period: 4 years
Marth et al.	**★**	**★**	**★**	**★**	**★**	**★**	**★**	**★**	**8**	Retrospective study; Clear inclusion and diagnostic criteria; Cup overhang measured using standardized and reproducible CT-based methods by blinded radiologists; limited adjustment for confounders was possible due to small IPI sample size (n = 16); low loss to follow-up; clear documentation of exclusions.
Odri et al.	**★**	**★**	**★**	**★**	**★★**	**★**	**★**	**★**	**9**	Retrospective study; Well-described inclusion criteria; 237 consecutive patient; Non-exposed group (ΔS < 6 mm) drawn from the same cohort and time period; Multivariate analysis adjusting for anteversion, inclination, and ΔS; age and sex not associated; Median follow-up 2 years is adequate for detecting persistent postoperative pain
Park et al.	**★**	**★**	**★**	**★**	**★★**	**★**	**★**	**★**	**9**	Retrospective study; IPT cases clearly defined, patients form a large cohort (1602 patients, controls drawn from the same cohort of THA patients without IPT, Adjusted for key confounders (sex, BMI, cup prominence, leg lengthening) in multivariate regression, exposures variables (cup prominence, leg lengthening, etc.) measured from radiographs by blinded observers, minimum 1 year follow-up
Ueki et al.	**★**	**★**	**★**	**★**	**★**	**★**	**★**	**★**	**8**	Retrospective study; Non-exposed patients (low protrusion group) from the same cohort; Screw protrusion length measured on standardized CT images with objective measurements; lack of multivariate analysis; minimum 12-month follow-up post-THA; single-center, single-surgeon series; limited sample size (n = 152 hips) may reduce generalizability.
Verhaegen et al.	**★**	**★**	**★**	**★**	**★★**	**★**	**★**	**★**	**9**	Retrospective study; Logistic regression adjusting for multiple risk factors (age, spine fusion, dysplasia, FAI, previous arthroscopy, bearing surface, etc.), Minimal loss to follow-up (2.2% lost); reasons for exclusions clearly documented; retrospective nature, and incomplete PROM data in ~40% of patients
Zhu et al.	**★**	**★**	**★**	**★**	**★**	**★**	**★**	**★**	**8**	Retrospective study; Consecutive series of dysplastic hips (Crowe II–IV) from a single institution, clear inclusion/exclusion criteria; Non-dysplastic hips from same time period and institution used as matched controls; no statistical adjustment for confounders; 12 patients lost to follow-up but interviewed by phone; follow-up completeness >85%; minimum 18 months follow-up (mean 45 months); variability in surgical techniques, and iliopsoas tendon release performed in 42% of dysplastic hips could confound true incidence.
Qiu et al.	**★**	**★**	**★**	**★**	**★★**	**★**	**★**	**★**	**9**	Retrospective study; diagnostic criteria well expressed, non-exposed cohort (IPI group) selected from the same institutional cohort, same surgical/diagnostic protocols, exposures measured via standardized radiographs and CT scans, multivariate regression adjusted for Dorr type, stem anteversion, and collar protrusion length; Minimum 1-year follow-up
Ueno et al.	**★**	**★**	**★**	**★**	**★★**	**★**	**★**	**★**	**9**	Retrospective study; Clear diagnostic criteria; controls selected from the same population (all THAs without IPI in same period); matched subset created for radiographic analysis; measurements conducted by blinded assessor; Multivariable logistic regression adjusted for age, BMI, sex, approach; matched controls for imaging analysis; No loss to follow-up for ≥ 2 years (569 THAs analyzed); clear documentation of exclusions;
Tamaki et al.	**★**	**★**	**★**	**★**	**★**	**★**	**★**	**★**	**8**	Retrospective study; diagnostic and inclusion criteria well documented; diagnosis based on clinical assessment only (no routine diagnostic injection). All radiographic evaluations repeated twice by 2 observers, each of whom was blinded to the results reported by the other. Asymptomatic hips from the same cohort served as controls; only univariate analysis; no multivariate adjustment due to small symptomatic samples (n = 24) blinded.

**Table 2:** Patients’ characteristics (/ = no data available).

Authors	Year	Type of Study	Level of Evidence	Geographic Provenience	Patients	Total Hips	Females	Males	Age (Years)	BMI	Mean Follow-Up
Buller et al.	2020	Retrospective cohort study	Level III	USA	518	559	296	222	63.6	/	55.9 months (24.0–126.0)
Hardwick-Morris et al.	2023	Cross-sectional imaging study	Level IV	UK	46	46	34	12	/	/	/
Kobayashi et al.	2023	Retrospective cohort study	Level III	Japan	138	138	11	27	65.0	23.7	48.0 months
Marth et al.	2023	Retrospective observational cohort study	Level III	Switzerland	220	220	116	104	62.7 ± 12.7	28.1 ± 5.7	24.2 ± 21.5 months
Odri et al.	2014	Retrospective cohort study	Level III	France	238	238	121	117	/	/	26.1 months (4.5–85.1)
Park et al.	2023	Retrospective cohort study	Level III	South Korea	1370	1602	790	580	55.0 (15.0–89.0)	24.6 (15.3–45.2)	48.0 months
Ueki et al.	2024	Retrospective cohort study	Level III	Japan	142	152	114	38	64.9	23.9 (5.3)	/
Verhaegen et al.	2022	retrospective case–control study	Level III	Belgium	1815	2120	1133	682	66.7	27.0 ± 5.0	43.2 ± 10.8 months
Zhu et al.	2019	Retrospective comparative cohort study	Level III	China	118 (DDH group) 115 (control group)	133 (DDH group), 126 (control group)	125 (DDH group) 119 (control group)	8 (DDH group) 7 (control group)	37.4 ± 10.8	23.2 ± 2.5	45.4 months (18.0–96.0)
Qiu et al.	2020	Retrospective observational study	Level IV	China	196	206	98	98	68.0	24.7	/
Ueno et al.	2018	Retrospective case–control study	Level IV	Japan	471	569	17 (IPI group) 419 (control group)	5 (IPI group) 128 (control group)	60.4 ± 11.5	23.2 ± 3.7	/
Tamaki et al.	2022	Retrospective cohort study	Level III	Japan	219	255	172	46	65.4	24.4	39.0 months

**Table 3:** Main findings and Risk factor for Ilepsoas Impingement (IPI) (/ = no data available).

Authors	Year	Total Hips	Surgical Approach	Bearing Surfaces	Stem Cementation	Collared/Collarless	Diagnosis of Ileopsoas Impingement	IPI Prevalence	Main Findings	Risk Factors
Buller et al.	2020	559	Direct Anterior	/	/	/	(1) Persistent anterior groin pain >6 weeks post-op worsened by resisted hip flexion or rising from a seated position (2) confirmed using false-profile radiographs showing anterior overhang and (3) response to ultrasound-guided diagnostic injection with local anesthetic.	5.7% (32/559 hips)	Incidence of AIPI: 5.7% (32/559 hips). Diagnosis made ~20 months post-op. 46.9% treated conservatively with resolution, 28.1% received ultrasound-guided corticosteroid injections, 21.9% underwent surgical intervention (arthroscopic psoas release or cup revision). Surgical resolution in most tenotomy cases, limited benefit from revision.	**Female sex (aOR 2.79)** **Cup-to-native femoral head ratio >1.2 (aOR 5.39)** **Anterior cup overhang ≥ 2 mm (aOR 5.53)** **Any measurable anterior overhang had aOR 7.07** **Cup malposition (inclination/anteversion) was not significantly associated with AIPI.**
Hardwick-Morris et al.	2023	46	Posterior	/	/	/	Surgeon 1: active hip flexion test in supine, no pain at rest, no pain with passive flexion of 10°, and pain with active flexion of 10° with a straight leg raise. Surgeon 2: pain at flexion in a bicycle test indicated anterior impingement between the iliopsoas and acetabular component leading to inflammation and pain from an apprehension test (extension and external rotation)	/	A novel simulation model detected significantly higher iliopsoas impingement in symptomatic vs. asymptomatic patients. Standing impingement (mean and maximum) was a stronger predictor of iliopsoas tendonitis than cup prominence (AUC 0.86 vs. 0.72). The simulation had 74–78% sensitivity and 91–100% specificity. This model can improve diagnosis and preoperative planning.	**Cup prominence > 6.5 mm (*p* = 0.002)** **Standing impingement > 0.04 mm (mean) o > 0.16 mm (maximum) (*p* = 0.002)**
Kobayashi et al.	2023	138	116 Posterior, 22 Anterolateral	CoP	30 Cemented; 108 cementless	/	(1) groin pain while ascending the stairs or getting in and out of a car; (2) groin pain with either resisted hip flexion in a seated position or straight leg raises in a supine position; (3) response to ultrasound-guided diagnostic injection with local anesthetic.	5.8% (8/138 hips)	Anterior position of the cup was related to symptomatic IPI and both axial and sagittal protrusion lengths at the most anterior margin of the cup. Anterior reaming and cup protrusion should be avoided as much as possible to prevent symptomatic IPI	**Sagittal Cup overhang (aOR = 1.77, *p* < 0.001)** **Axial Cup overhang (aOR = 1.16, *p* = 0.007)** **Oversized Cup (aOR = 1.26, *p* = 0.192)**
Marth et al.	2023	220	Direct Anterior	/	/	/	(1) clinical signs (groin pain, positive Thomas test), (2) imaging findings (MRI or ultrasound showing iliopectineal bursitis or iliopsoas tendinopathy), in some cases, (3) confirmation via pain relief after fluoroscopy-guided corticosteroid injection into the iliopsoas tendon sheath	7.3% (16/220 hips)	Cup overhang (CO) present in 10.4% of cases. IPI diagnosed in 7.3% (16/220 hips). Patients with IPI had significantly higher ODc (corrected overhang distance) (12.5 ± 4.3 mm) compared to non-IPI group (7.4 ± 3.6 mm; *p* = 0.002). A cutoff of ODc ≥7.5 mm had sensitivity of 90.9% and specificity of 77.8% for detecting IPI.	**Cup overhang (OR = 35.20, *p* < 0.001)**
Odri et al.	2014	238	/	MoP, CoP; CoC (not specified in which proportion)	/	/	Anterior groin pain worsened by active flexion and relieved with passive flexion	4.6% (11/238 hips)	Cup oversizing and low inclination significantly associated with AIPI. Anteversion not significant in multivariate analysis. Patients with AIPI had a significantly higher implanted cup size ICS (*p* = 0.04), a significantly higher difference between the implanted cup size and the native femoral head size (ΔS) (*p* = 0.001) and a significantly lower inclination (*p* = 0.03) compared to the control group.	**Cup oversizing (ΔS) ≥ 6 mm: aOR = 26.00 (*p* < 0.001)** **Lower cup inclination (*p* = 0.03)** **Anteversion not significant**
Park et al.	2023	1602	Anterolateral	/	all cementless	all collarless	(1) anterior groin pain lasting more than 3 months; (2) pain triggered by daily activities with hip flexion; (3) pain reproduced by active SLR and aggravated by resisted SLR; (4) pain improved after injection of lidocaine and corticosteroids into the iliopsoas tendon sheath	3.3% (53/1602 hips)	Patients with IPT had greater leg lengthening (12.3 versus 9.3 mm; *p* = 0.001) and higher prevalence of antero-inferior cup prominence (5.7 versus 0.4%; *p* = 0.002). There was no significant difference in inclination, anteversion, and horizontal offset change between the two groups. In multivariate analyses, greater leg lengthening, prominent acetabular cup, women, and higher body mass index were associated with IPT.	**Leg lengthening (aOR 1.06, *p* = 0.018)** **Anterior cup prominence (aOR 15.43, *p* = 0.002)** **Female sex (aOR 2.56, *p* = 0.012)** **BMI (aOR 1.15, *p* < 0.001)**
Ueki et al.	2024	152	Anterolateral	/	all cementless	/	(1) clinical test (pain during active straight-leg raise), (2) CT showing screw protrusion, and (3) pain relief ≥3 points on NRS after xylocaine + steroid injection into iliopsoas at ilio-pubic eminence	6.5% (10/152 hips) in a cohort with screw protrusion	The threshold for screw protrusion length was identified as 6.4 mm. Patients in the high protrusion group exhibited significantly larger area and lower Hounsfield Unit values of the iliopsoas muscle. In addition, the high protrusion group revealed significantly lower scores (total, pain, movement, mental).	**Screw protrusion > 6.4 mm (*p* < 0.001)**
Verhaegen et al.	2022	2120	Direct Anterior	CoC 1188 CoP 932	105 cemented, 2015 cementless	1225,Collared; 869 Collarless	(1) persistent postoperative groin pain, triggered by hip flexion; (2) decrease in pain after fluoroscopy-guided iliopsoas tendon sheet injection with xylocaine and corticosteroid.	2.2% (46/2120 hips)	Younger age and presence of a spine fusion were the significant predictors of IPI. Higher incidence of psoas pain in with ceramic-on-ceramic (CoC) bearing surface in comparison to patients with ceramic-on-polyethylene (CoP) bearing. Patients with IPI reported more low back pain (OR 4.7; *p* < 0.001) and greater trochanteric pain (OR 5.2; *p* = 0.011)	**Younger age (*p* < 0.001)** **Spine fusion (OR 4.6, *p* = 0.008)** **Previous hip arthroscopy (OR 9.6; *p* = 0.006)**
Zhu et al.	2019	259	Posterior	CoC 128, CoP 2, MoP 3.	all cementless	all collarless	(1) Groin pain during hip flexion; (2) Pain/tenderness with resisted flexion; (3) Pain relief after ultrasound-guided peritendinous injection of corticosteroid and anesthetic	1.5% (2.6% DHH group/0.8% control group)	No significant difference in incidence of iliopsoas tendinitis between DDH (2.6%) and control (0.8%). Higher prevalence of anterior overhang (30.8%), protruding screws (24.8%), and leg lengthening (3.6 cm) in DDH group. Newly proposed mechanism: anterior instability due to excessive cup anteversion causing iliopsoas irritation (combined antiversion for IPI group≃60°). Surgical iliopsoas release in 42.1% of DDH patients helped prevent contracture complications.	**Protruded acetabular screws (*p* < 0.05)** **Anterior cup overhang (*p* < 0.05)** **Excessive leg lengthening (*p* < 0.05)** **Excessive anteversion causing anterior instability** **No single factor independently predicted IPI**
Qiu et al.	2020	206	Posterior	/	all cementless	all collared	(1) persistent anterior groin pain after 3 months postoperatively; (2) anterior groin pain triggered by active hip flexion and active flexion against resistance with pain continuing from 30 to 70° flexion; (3) pain increased in active internal rotation and reduced in external rotation; and (4) pain improved after injection with lidocaine and steroid into the iliopsoas tendon sheath under the guidance of ultrasound	7.3% (15/206 hips)	Increased stem anteversion (19.1° vs. 15.2°, *p* < 0.001) and collar protrusion length (CPL: 2.6 mm vs. –0.5 mm, *p* < 0.001) were independent risk factors. Dorr type C femur is more frequent in the IPI group (60% vs. 14.7%, *p* < 0.001), but not a significant predictor in the regression model.	**Increased stem anteversion (OR = 1.74, *p* = 0.001)** **Collar protrusion length (OR = 13.89, *p* = 0.001)** **Dorr type C proximal femur (*p* < 0.001)**
Ueno et al.	2018	569	Posterior 498; Anterolateral 71	/	44 cemented; 525 cementless	/	(1) Anterior groin pain reproducible during active or passive hip flexion or extension. (2) Significant pain relief following a diagnostic injection of xylocaine and corticosteroid into the iliopsoas tendon sheath.	3.9% (22/569)	Axial protrusion ≥12 mm (aOR=17.13), sagittal protrusion ≥4 mm (aOR=8.23) significantly predicted IPI. Anterolateral surgical approach increased risk (OR = 4.20). Greater native acetabular version and lower cup anteversion/inclination were associated with more protrusion. Sensitivity/specificity for thresholds were high (72–91%)	**Anterolateral approach: OR = 4.20 (*p* = 0.002)** **Axial protrusion ≥ 12 mm: aOR=17.13 (*p*=0.002)** **Sagittal protrusion ≥ 4 mm: aOR=8.23 (*p*=0.018)**
Tamaki et al.	2022	255	Anterolateral	/	cementless	collarless	(1) persistent anterior groin pain continuing for at least 3 months; (2) anterior groin pain triggered by active hip flexion or passive hip extension; (3) pain increased in active internal rotation and reduced in external rotation	11.0%	Cup protrusion significantly higher in the IPI group (4.7 mm vs. 1.4 mm, *p* = 0.001). Hip flexion angles (pre and post-op) were significantly greater in the IPI group (*p* = 0.013, *p* = 0.006). Threshold for cup protrusion: 3.9 mm (sensitivity = 0.89, specificity = 0.63). Posterior pelvic inclination may mitigate symptoms.	**Cup overhang (*p* = 0.001)** **Greater post-operative hip flexion angle (*p* = 0.013);** **Posterior pelvic inclination may reduce IPI risk despite protrusion**

**Table 4:** Effect size of risk factor for Iliopsoas Impingement (IPI) (/ = no data available).

Risk Factor	Study (Author)	Odds Ratio (OR)	95% CI	*p*-Value	Significant?
**Anterior cup prominence/overhang**	Buller et al.	7.07	2.52–19.78	<0.001	yes
	Hardwick-Morris et al.	/	/	0.024	yes
	Kobayashi et al.	Sagittal (1.77); Axial (1.16)	Sagittal (1.29–2.41) Axial (1.03–1.29)	Sagittal (<0.001) Axial (0.007)	yes
	Marth et al.	35.20	10.53–117.73	<0.001	yes
	Park et al.	15.43	3.75–63.47	0.002	yes
	Zhu et al.	/	/	<0.001	yes
	Ueno et al.	Sagittal (8.23); Axial (17.13)	Sagittal (1.44–46.94) Axial (2.78–105.46)	Sagittal (0.018) Axial (0.002)	yes
	Tamaki et al.	/	/	0.001	yes
**Cup-to-native femoral head ratio >1.2**	Buller et al.	5.39	1.60–18.40	0.007	yes
**Standing mean impingement** **(> 0.04 mm)**	Hardwick-Morris et al.	/	/	0.018	yes
**Cup oversizing**	Kobayashi et al.	1.26	0.89–1.66	0.192	no
	Odri et al. (ΔS ≥ 6 mm)	26.00	6.30–108.00	<0.001	yes
**Lower cup inclination values**	Odri et al.	/	/	0.03	yes
**BMI**	Park et al.	1.14	1.07–1.21	<0.001	yes
**Leg lengthening**	Park et al.	1.06	1.03–1.11	0.018	yes
	Zhu et al.	/	/	< 0.001	yes
**Screw protrusion**	Ueki et al.	/	/	< 0.001	yes
	Zhu et al.	/	/	< 0.001	yes
**Female sex**	Bullet et al.	2.79	1.18–6.56	0.020	yes
	Park et al.	2.56	1.36–4.82	0.012	yes
**Younger age**	Verhaegen et al.	/	/	< 0.001	yes
**Spine fusion**	Verhaegen et al.	4.60	1.60–13.40	0.016	yes
**Previous hip arthroscopy**	Verhaegen et al.	9.60	2.60–34.30	0.002	yes
**Increased stem anteversion**	Qiu et al.	1.74	1.25–2.43	0.001	yes
**Collar protrusion length**	Qiu et al.	13.89	3.14–62.50	0.001	yes
**Anterolateral approach**	Ueno et al.	4.20	1.68–10.49	0.002	yes
**Post-operative hip flexion angle**	Tamaki et al.	/	/	0.013	yes
